# Evaluating the impact of NHS strikes on patient flow through emergency departments

**DOI:** 10.1136/emermed-2024-214452

**Published:** 2025-12-25

**Authors:** Alex Garner, Quin Ashcroft, Dale William Kirkwood, Vishnu Chandrabalan, Hedley Emsley, Suzanne M Mason, Nancy Preston, Jo Knight

**Affiliations:** 1 Lancaster Medical School, Lancaster University Faculty of Health and Medicine, Lancaster, Lancashire, UK; 2 Lancashire Teaching Hospitals NHS Foundation Trust, Preston, Lancashire, UK; 3 School of Health and Related Research, University of Sheffield, Sheffield, UK; 4 International Observatory on End of Life Care, Lancaster University, Lancaster, UK

**Keywords:** Operations, Routinely Collected Health Data, Efficiency, Observational Study, Statistics

## Abstract

**Background:**

Since December 2022, the National Health Service (NHS) has experienced large-scale strikes by staff. The NHS cancels approximately 12 million elective care appointments each year, and around 1 million elective appointments were cancelled due to strikes between 2022 and 2024. During strikes, emergency care is prioritised, and it has been claimed that emergency departments (EDs) run ‘better than usual’. The aim of this study was to investigate changes in patient flow into hospitals through the ED during the strike periods.

**Methods:**

Cox proportional hazards modelling was applied to data from two different EDs in the north-west of England to model time between patient arrival at the ED and their subsequent admission. Systematic (linear temporal trend, yearly seasonality, daily seasonality, weekends, ED ‘heat’) and patient/presentation-level factors (urgency, service referred to, patient age, ethnicity and gender) were controlled for. The impact of different striking professions on patient time to admission was investigated using HRs, where a higher HR indicated faster admission.

**Results:**

Over the analysis period, we observed 61 separate strike days: 40 junior doctor strike days, 11 nursing days, 10 consultant days and 7 ambulance days. Junior doctor and consultant strikes coincided on 4 days. For the type 1 ED, median time to see a clinician was similar on strike and non-strike days (median 2 hours 27 min on strike days (IQR: 1 hour 2 min to 4 hours 53 min), 2 hours 27 min on non-strike days (IQR: 1 hour 5 min to 5 hours 14 min)). Patients were admitted through the ED more quickly on both the junior doctor and consultant strike days compared with non-strike days (HRs: 1.12, 1.28, both p≤0.001). This increased flow was only seen while consultants were striking in the type 2 smaller ED.

**Conclusions:**

These findings suggest that the improved patient flow observed on strike days could be driven by the additional inpatient capacity created through the postponement of elective care. This result indicates that NHS hospital systems could potentially be adjusted to enhance turnaround times and reduce ED crowding.

**WHAT IS ALREADY KNOWN ON THIS TOPIC**

- The National Health Service experienced a number of staff strikes in recent years by junior doctors, consultants and other staff, with anecdotal reports of faster flow through the emergency department (ED) for admitted patients on strike days.
- Similar effects have been seen for doctor strikes in other countries.
- The improvement has been variously attributed to consultant-led care, patients staying away and cancelled elective surgeries.

**WHAT THIS STUDY ADDS**

- This study identifies significant improvements in flow of admitted patients into the hospital during the strikes, when accounting for patient and systematic factors.
- The greatest improvement in time to admission was on days of consultant strikes and days of junior doctor strikes.
- Notably, median time to see a physician in the ED was similar on both strike and non-strike days.

**HOW THIS STUDY MIGHT AFFECT RESEARCH, PRACTICE OR POLICY**

- The observed improvements in patient flow suggest that although strike-day service is not sustainable, elements of strike preparation such as increasing inpatient capacity could be applied to improve the efficiency of ED operations.

## Introduction

 Since December 2022, the National Health Service (NHS) has experienced large-scale strikes over pay and working conditions by junior doctors, consultants and other staff.^1^ In June 2023, nurse strikes in England ended due to low voter turnout. In April 2024, consultants agreed to end their strikes with a new pay deal.^2^ Resident doctors followed suit in September 2024^3^ but have subsequently started striking again. By the end of 2023, the NHS estimated that the costs of the strike amounted to around £1.5 billion.^4^

During strike periods, a substantial proportion of routine care was rescheduled and approximately 1 million appointments were cancelled between February 2022 and January 2024.^5 6^ This contributed to the approximately 12 million elective care cancellations each year between 2021 and 2023.^7^ Cancellations impact patient outcomes, with a survey by *Healthwatch* finding that 66% of people with cancelled care for any reason during 2023 said it had impacted their lives.^8^

Emergency care provided by the NHS is protected and prioritised during strikes. Non-striking staff are often drafted in to cover the emergency department (ED).^6^ The process by which patients are admitted to hospital through the ED typically does not change during strikes. The core elements of the process—referral to a specialty, clerking by that specialty and finding an appropriate bed—remain; only the mix of staff making the decisions is altered.

Evidence from previous strikes has shown that strikes are associated with fewer emergency attendances, leading to less crowding in the EDs.^9^ ED crowding is generally associated with an increased risk of in-hospital mortality, longer times to treatment of patients with certain conditions and a higher probability of leaving the ED against medical advice or without being seen.^10^ Studies conducted under non-strike conditions suggest that the primary cause of ED crowding is exit block (or access block).^11 12^ Exit block is defined as when ‘patients in the ED requiring inpatient care are unable to gain access to appropriate hospital beds within a reasonable time frame’.^13^ The clinical risks of increased waiting times are well studied; for example, worse adherence to guidelines in certain types of myocardial infarctions^14^ and time to antibiotics in cases of community-derived pneumonia.^15^

In a commentary in the *British Medical Journal* in 2023, the President of the Royal College of Emergency Medicine wrote that during some of the strikes ‘in the emergency department everything works better than usual’.^16^ The commentary pointed to streamlined decision making by consultant-led teams, lower attendances due to media coverage and cancelled elective surgeries as possible reasons for this improved performance. Some studies outside the UK indicate that junior doctor strikes are correlated with lower patient waiting times, supporting the hypothesis about the impact of streamlined decision making by consultants.^17^

We hypothesise that patient admittance from the ED is expedited on strike days (reflecting improved patient flow) and aim to provide quantitative evidence to support NHS consideration of changes that can affect ED operations. In this study, we analysed data on time spent in two EDs in the north-west of England. We applied a Cox proportional hazards model to examine the time from ED arrival to patient admission, controlling for other influential variables and evaluating the impact of strike action.

## Methodology

### Data

The data set structure was based on the NHS Emergency Care Data Set specification, a national specification for data sets from NHS EDs, set by NHS England and the Royal College of Emergency Medicine.^18^ The data included attendances to two EDs in the north-west of England between January 2022 and April 2024, giving time of arrival, time spent in ED, investigations performed and other demographic and diagnostic information. Data were excluded for any patients who had opted out of their NHS data being used for research.

Both EDs are operated by the same Trust. ED1 is a 24-hour, full-service department with a major trauma service, averaging around 50 000 attendances per year since 2022. ED2 is an adults-only minor injuries unit with limited opening hours, averaging around 25 000 attendances per year since 2022. Patients with major injuries presenting to ED2 are transferred to ED1. The EDs are analysed separately; this paper focuses on the effects of strike action at the major ED1, as the results are likely to be more generalisable to other EDs. Full results for ED2 may be found in the supplementary materials, with key findings discussed in the main paper.

### Outcome

The outcome for this analysis was time spent in ED for patients admitted to hospital directly from their ED attendance. This was used as a proxy measure for patient flow through the ED. This measure was calculated based on the time between patient arrival and departure from the ED. The departure time was defined according to the NHS data dictionary as the time a patient is discharged or transferred from the emergency care attendance to a ward.^19^ We used only the subset of ED attendances that are transferred to a ward, excluding any patients who were discharged or died before admission because such patients do not shed light on flow through the hospital. From this point, we refer to this as the *time in ED Given Subsequent Admission (time in EDGSA)* where there are multiple mentions in a section.

Time in EDGSA can be thought of as *time to event* data that can be used in a Cox proportional hazards model. In this case, since the endpoint of interest—admission—is an outcome where earlier occurrence is desirable, a higher HR indicates that admitted patients spend less time in ED.

The findings assume that admission processes do not change during strike days for reasons elucidated in the introduction.

### Exploratory testing for strike day changes

We used Kolmogorov-Smirnov two-sample tests to assess the potential changes in number of admissions per day, proportion of attendances admitted to a ward, and time taken to be seen by a clinician during strike days and non-strike days over the study period.

### Kaplan-Meier exploratory analysis

We used Kaplan-Meier plots to show the proportion of patients remaining in the ED over time, measured in hours since arrival. The plots were used to investigate the empirical differences between categorical factors that we hypothesise could impact patient flow through the ED, including service referred to, urgency, ethnicity, gender and striking profession. CIs are calculated using Greenwood’s formula to estimate variance of the estimate at each time point.^20^ All categorical variables were used in the model as all demonstrated visual differences in the curves according to the different factors.

### Cox proportional hazards modelling

Cox proportional hazards models were used to produce a semiparametric regression model fitted to time in ED given subsequent admission—fitted using the *lifelines* package in Python.^21^ We created two models (for each of ED1 and ED2), each with five strike groups of interest to investigate the impact of the following strikes: *junior doctor strike*, *consultant strike*, *both junior doctor and consultant strike*, *nurse strike*, *ambulance strike*. Strike day categorisations are found in online supplemental table S1.

We also controlled for variables that could influence patient flow on strike days, or factors that may alter an individual’s time spent in ED. Further details of how these variables were constructed are found in the ‘Model Covariates’ section in the online supplemental materials. Variables accounted for in the Cox-regression model are:

- ED factors
  - Linear time effect
  - Seasonal effects—harmonic pair
  - Time of day—harmonic pair
  - Weekend—binary variable
  - ‘Heat’—measure of number of patients in the ED scaled by urgency of those patients
- Patient presentation factors
  - Urgency of presentation (This is a variable defined by the NHS dictionary as ‘the category assigned to a PATIENT as a result of an initial assessment by medical or nursing staff in an Accident and Emergency Department’.)
  - Referred to service
- Patient demographic factors
  - Age
  - Ethnicity
  - Gender.

We used the Cox proportional hazards model with SEs calculated robustly via a bootstrapping method.^22 23^ HRs were calculated for each variable in the model, including the strike variable. The magnitude of the HRs for junior doctor strikes and other strikes was tested at an α=0.005 significance level—Bonferroni adjusted for the five strike types (compared with no strike) at each ED (10 tests). We also presented raw p values to allow accurate interpretation as the Bonferroni adjusted level may be conservative due to possible correlation in the data. The proportional hazards assumption was assessed using log-log plots of empirical time in the ED given subsequent admission curves of the variables and Schoenfeld residuals of the fitted Cox models.^22^

### Additional analysis

The heat variable at the time of arrival is an upstream variable that impacts time in ED. However, it is also contributed to by the patients when they arrive, meaning there is a risk of reverse causality. To investigate the impact of the ‘heat’ variable, an additional analysis was undertaken without this variable in the model.

### Patient and public involvement

ED staff were involved in the initial formulation of this project’s research question. No patients or members of the public were involved in this research.

## Results

The results presented in the main paper relate to ED1 and results from ED2 are presented in the online supplemental materials section ‘ED2 Analysis’ (online supplemental table S3-S5 and figure S14-S15). There were 174 961 emergency attendances in the study period, 119 553 were to ED1. Of those, 49 165 (41%) resulted in an admission to the hospital. Observations where hospital admission status, referred to service or urgency were missing in the data were not included, resulting in 44 229 admissions in the sample for investigation.

Over the analysis period, we observed 61 separate strike days; the first was 15 December 2022. There were 40 junior doctor strike days, 11 nursing strike days, 10 consultant strike days and 7 ambulance strike days. A junior doctor and consultant strike coincided on 4 days (online supplemental table S1). The cohort selection flow diagram can be found in online supplemental figure S1. The patient demographics overall and specifically for strike days can be viewed in table 1.

**Table 1:** Demographic mix of patients overall and filtered for strike days, by individual. An individual can appear in both groups if they attend on a strike day and a non-strike day during the study period

Characteristic	Grouping	Count of individuals for the entire period	% of overall cohort	Strike day count of individuals*	% of strike day cohort
Age, years	(0, 18)	3716	*12*	263	*12*
	(18, 25)	1363	*4*	92	*4*
	(25, 30)	1081	*3*	88	*4*
	(30, 45)	3560	*11*	250	*12*
	(45, 65)	6498	*21*	415	*19*
	(65, 80)	7854	*25*	538	*25*
	≥ 80	7527	*24*	486	*23*
Gender	Female	15 550	*49*	≈1070	*50*
	Male	16 034	*51*	≈1060	*50*
	Not known	15	*0*	< 5	*0*
Ethnicity	White	27 460	*87*	1834	*86*
	Asian or Asian British	1807	*6*	116	*5*
	Black or black British	313	*1*	23	*1*
	Mixed	311	*1*	19	*1*
	Other ethnic groups	329	*1*	27	*1*
	Not stated	1379	*4*	113	*5*

* Some counts are approximate, preventing small number disclosure and protecting the identity of individuals in the study.

### Comparison of attendances between strike and non-strike days

There were 3219 attendances During the 61 strike days (daily mean 52.8, SD 7.4), and 41 010 attendances during the 760 non-strike days (daily mean 54.0, SD 8.0) at ED1. When comparing the attendances per day using the Kolmogorov-Smirnov test, we found no significant difference between strike days and non-strike days (p=0.213). We found no significant difference between the proportion of attendances admitted on strike days (p=0.093). See table 2.

**Table 2:** Comparison of daily attendances and admissions during strike and non-strike periods. SDs are included in brackets

	Mean attendances per day	Proportion of admissions (%)	Median time in ED, hours and minutes (IQR)	Median time to see a clinician, hours and minutes (IQR)
Non-strike days (n=760)	52.8 (7.4)	41.8 (5.2)	18 hours 4 min (8 hours 44 min to 28 hours 47 min)	2 hours 27 min (1 hour 2 min to 4 hours 53 min)
Strike days (n=61)	54.0 (8.0)	41.2 (4.9)	13 hours (6 hours 58 min to 23 hours 34 min)	2 hours 27 min (1 hour 5 min to 5 hours 14 min)
Kolmogorov-Smirnov value of p	0.213	0.093	<0.001	<0.001

ED, emergency department.

### Average outcomes

**Figure 1:**
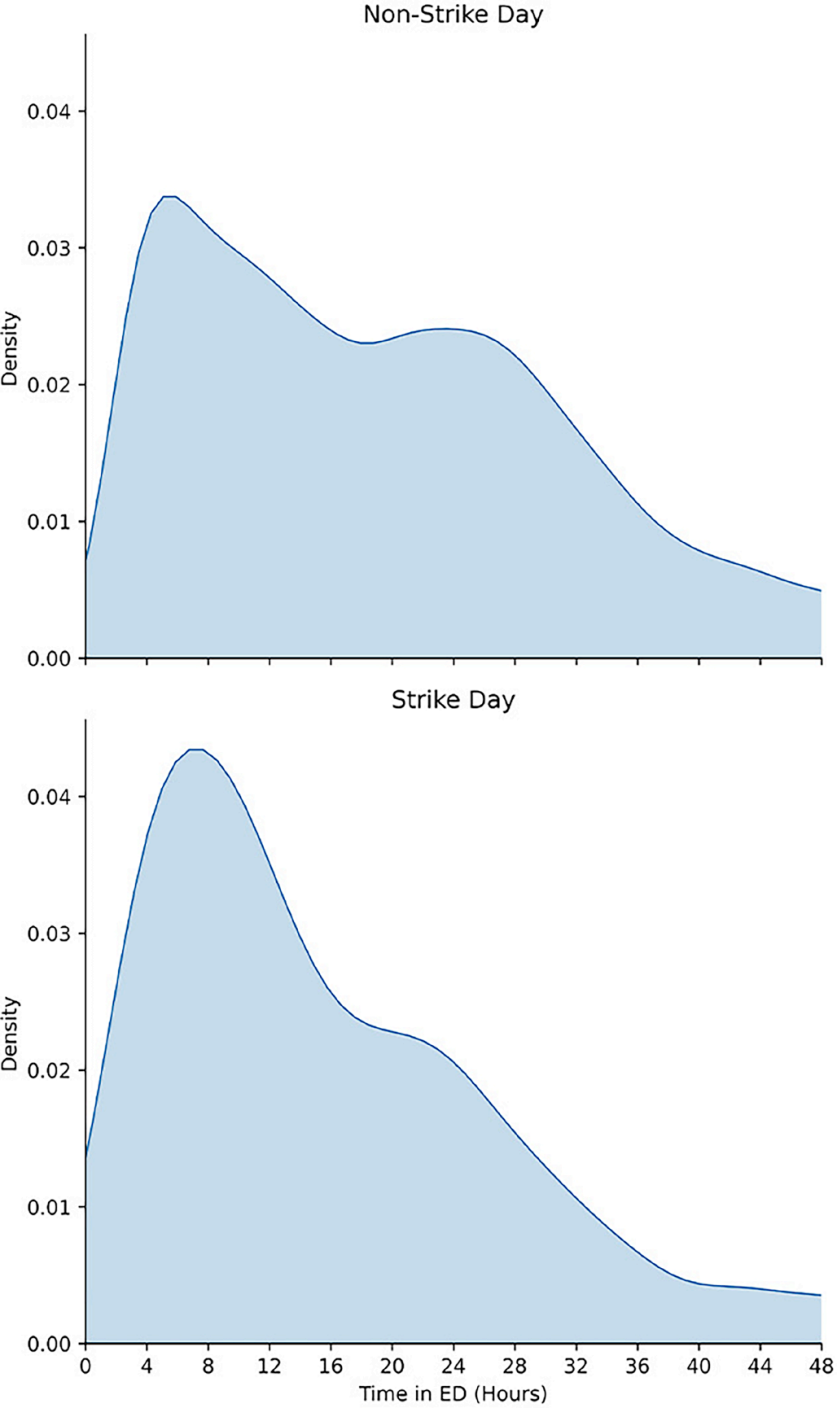
Normalised density of time in the emergency department (ED) given subsequent admission, separated by strike and non-strike days. Kernel density estimation is used to normalise the difference in group sizes.

The distributions of patients’ time in ED given subsequent admission separated by strike days and non-strike days are found in figure 1; a histogram of the time in EDGSA is found in online supplemental figure S2. Over the entire period of observation, the median time in EDGSA was 18 hours 4 minutes (IQR: 8 hours 44 min to 28 hours 47 min). The median time in EDGSA on a strike day was 13 hours (IQR: 6 hours 58 min to 23 hours 34 min) (see table 2). There was a high rate of admissions immediately before the 4-hour mark, corresponding to patients being admitted immediately before the 4-hour target time set by the government^24^ and skewing the distribution of time in EDGSA.

The median time to see a clinician on a strike day was 2 hours 27 min (IQR: 1 hour 2 min to 4 hours 53 min). The median time to see a clinician on a non-strike day was 2 hours 27 min (IQR: 1 hour 5 min to 5 hours 14 min). The KM test found a significant difference between the two groups (p=2.273e-5); however, this significant result is likely due to the large sample size (every admission during the study period), as it can be seen from the medians and the box plot of distributions in online supplemental figure S3 that the two groups are very similar.

### Kaplan-Meier exploratory analysis

**Figure 2:**
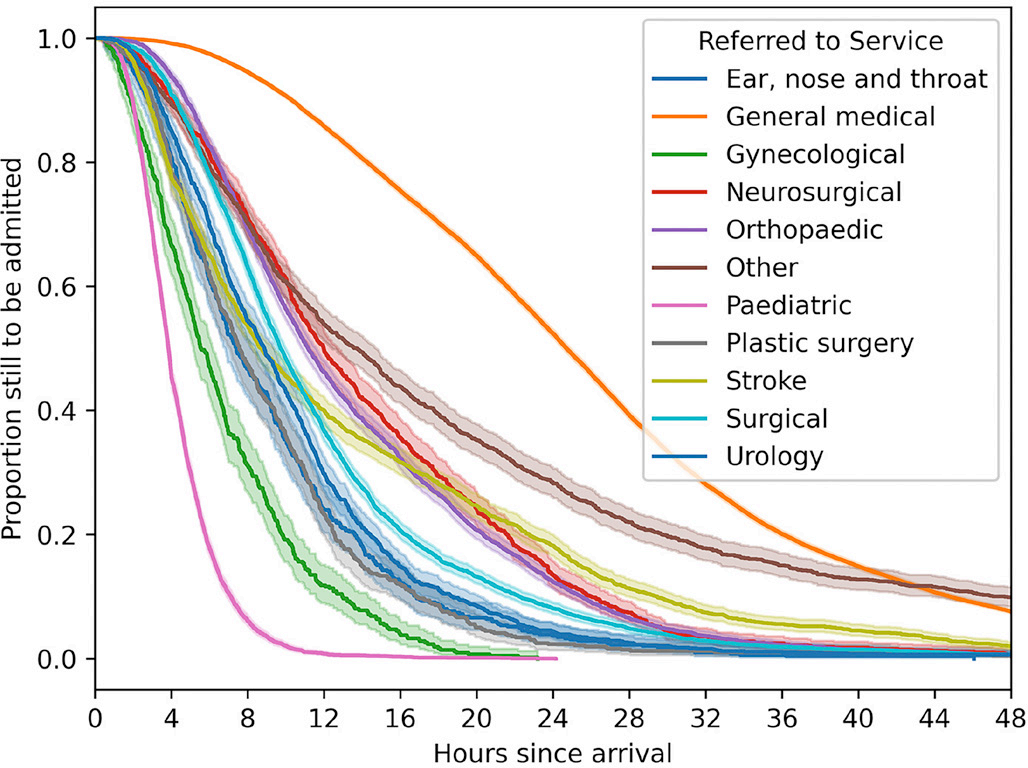
Kaplan-Meier curves of admitted patients’ time in the emergency department (ED) given subsequent admission, separated by the referred to service they are admitted to. The shaded area around each line represents the 95% CI for the KM estimate at each point. These CIs are very small for paediatrics and general medicine and are therefore not easily visible on the plot.

The Kaplan-Meier exploratory analysis demonstrated different rates of admission across the strike types (online supplemental figure S7). It was also used to explore the differences in other categorical variables regardless of strike conditions; variables included urgency, service referred to, ethnicity and gender. Figure 2 shows that the curves were a similar shape across specialties (with the exception of stroke services) but rates of admission varied. Patients referred to Paediatrics had the shortest time in ED and those referred to General Medicine had the longest. The full list of services included in this category can be found in the supplementary materials (*referred to* service *categories* section). The Kaplan-Meier curves for other categorical explanatory variables (urgency, gender and ethnicity) similarly showed a variation in rates (online supplemental figures S4—S6).

### Cox proportional hazards modelling results

Coefficients and HRs (for earlier admission) for the strike types are found in table 3. As noted in Methods, while a higher HR refers to a higher likelihood of being admitted into the hospital, in this case, since we only investigate patients who were admitted, the higher HR means patients were more likely to be admitted more quickly.

**Table 3:** Coefficients and HRs for the strike variables in the Cox proportional hazards model. Here, higher hazard refers to a higher likelihood of being admitted into the hospital. Since we only investigated patients who were admitted, higher hazard is a positive outcome that means patients were more likely to be admitted more quickly

Strike type	Coefficient	HR	95% CI		P value
Baseline—no strike			–	–	–
Junior doctor strike	0.117	1.124	1.059	1.194	<0.0001*
Consultant strike	0.249	1.283	1.113	1.478	0.0006*
Consultant and junior doctor strike	0.099	1.105	0.918	1.33	0.293
Nurse strike	0.115	1.122	1.024	1.229	0.0134
Ambulance strike	0.073	1.076	0.947	1.222	0.260

* Statistically significant.

The extended table for all variables is found in online supplemental table S2. Forest plots for all variables are found in online supplemental figure 13.

**Figure 3:**
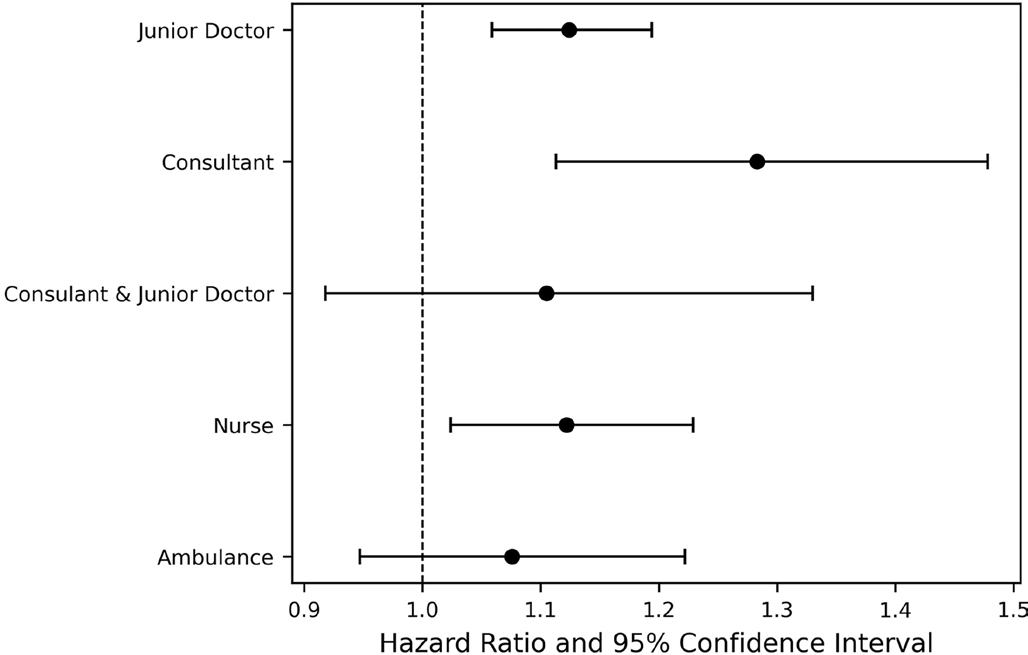
Forest plot demonstrating HRs and CIs of the impact of strikes on patients’ time in the emergency department (ED) given subsequent admission compared with the baseline of no strike—higher hazard implies patients moving through the ED faster.

**Figure 4:**
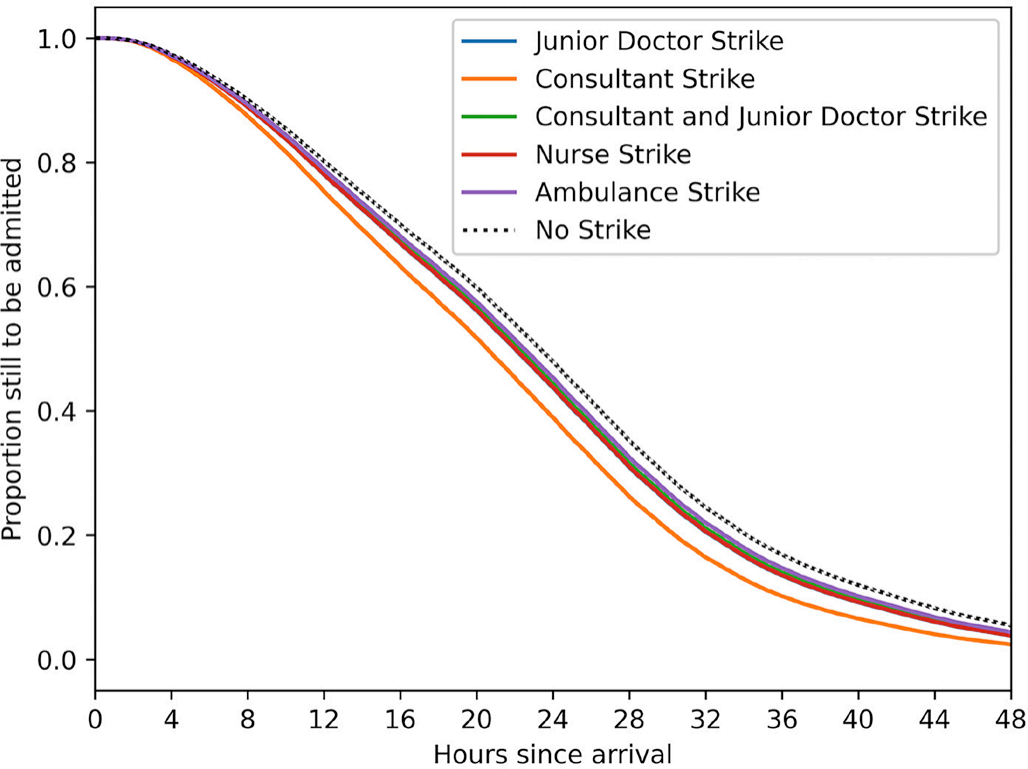
Fitted Cox-regression time in the emergency department (ED) given subsequent admission curves for each of the different strike types in the analysis.

The results suggest that consultant and junior doctor strikes were the two strike types that impacted a patient’s time in EDGSA to a statistically significant degree. The largest effect is for consultant (only) strikes, with an HR of 1.28. The next largest effect was on junior doctor (only) strike days, HR: 1.12, both suggesting that patients move through the ED into the hospital more quickly on these strike days, leading to shorter time in EDGSA. All other strike types have a point estimate in favour of improved flow but are not statistically significant. The Cox proportional hazards HRs and CIs for all the strike types are shown in forest plots in figure 3. The fitted Cox proportional hazards time in EDGSA model curves for each strike type can be found in figure 4.

The Schoenfeld residuals against transformed time tests indicated that five of the variables included in the model significantly deviated from proportional hazards. Inspected visually, these were minor deviations that were likely to be statistically significant due to large sample sizes. Results from these tests are found in online supplemental figures S8–S12 and are considered further in the *Discussion* section. The findings from the model without the heat variable are present in the online supplemental materials section ‘Additional Analysis’; they are more significant and have a greater magnitude, but we consider the main analysis more robust because although there may be a small influence of heat on time in ED, it is an important upstream covariate that needs to be adjusted for in the analysis.

## Discussion

We provide quantitative evidence of the impact of the NHS strikes on patient flow into the hospital through the ED. We find a shorter time to admission from ED for admitted patients on junior doctor and consultant strike days, when controlling for ED and patient factors suggesting improved patient flow on strike days compared with non-strike days. This is despite the fact that the number of attendances and the proportions of patients admitted was not different between strike and non-strike days and time to see a clinician was not visibly different between the strike and non-strike days.

The authors do not suggest that healthcare worker strikes are positive overall. During strikes, the hospitals were running unsustainable practices.^16^ The issues of cancelled care and rising costs to the NHS have been raised in the introduction, and cancelled elective care can lead to further ED attendances down the line. Furthermore, ED staff are experienced in making decisions whether to admit patients quickly and safely, and we do not investigate whether the decisions to admit in this data set were appropriate. However, we identify that there was no increase in the proportion of patients admitted during specific strike periods. These observed changes imply that improvements to patient flow through EDs may be possible, leading to reduced patient time spent in the ED.

The two strike types that were found to be significantly associated with patient time in the ED given subsequent admission were those that impacted clinical decision makers (consultants and junior doctors). It is likely that specific preparations are necessary when strikes include these professions. ED doctors can strike, but their action can be more limited because emergency care is prioritised during strikes. One of the factors influencing faster patient flow through the ED could be additional capacity for patients admitted from the ED due to the large cancellation of elective activity in hospitals reported across the country.^25^ This may also be influenced by increased discharges of inpatients leading up to strike days. Specialists also often have additional capacity to deal with emergency patients.^26^ Reduction of elective care may also free up other hospital capacity, such as in the intensive care unit and diagnostic services. The Royal College of Emergency Medicine reported in January 2024 that hospital bed occupancy rates were at the ‘unsafe’ level of 93%.^27^ Improved flow out of ED, with no change in time to see a clinician, during strikes supports the hypothesis that exit block is the primary driver of ED crowding—indicating improved flow of patients out of hospital will likely improve waiting times in the ED.

Our findings agree with prior literature that implies a reduction in patient wait time during strike periods globally.^28^,^30^ However, findings are inconsistent and depend on the country’s healthcare system and the strike in question.^17^ Evidence from previous NHS strikes shows the negative impact on services and patient outcomes, but does not specifically investigate EDs.^31 32^

An additional finding of the paper is the large variation in time in EDGSA admission curves for the different services referred to. Many of the differences between the different categories and the baseline of ‘orthopaedics’ in the fitted model are statistically significant overall (and vary between each other). The differences in time to event time in EDGSA curves between referred to services demonstrated in figure 2 are likely due to hospital flow structures. Flow and capacity issues that contribute to exit block vary between patient destinations. Due to the different case mix in hospitals, this would likely impact results if this is not taken into account.

### Limitations

The findings in this paper agree with other international literature on patient flow and ‘waiting time’ during strikes. The impact of strikes will likely vary by hospital and needs to be confirmed in other settings, for example, in a setting where the admittance rate in the ED is closer to the national level, our data showed an admission rate of 41% where the national rate is about 30% according to the NHS *Getting It Right First Time* report.^33^

NHS data are not recorded for research and are not always clean data. For example, there are peaks in admission just before the 4-hour mark which are likely driven by the behaviour of the staff; therefore, they don’t fully reflect healthcare requirements. Such irregularities affect all data and are unlikely to influence the results.

The only strike data we have are the dates of each type of strike (by profession of striker); we therefore assume other factors are equal. For example, that striking groups cover each other’s strikes equally. Employment of locum doctors, differences in staff volume or certain subgroups not striking during this time would not be accounted for and may skew results.

Our Cox proportional hazards methodology has some limitations. The model falls short of completely satisfying the proportional hazards assumption. The assumptions were tested using Schoenfeld residuals and log-minus-log plots to assess validity of the results. However, it is well documented that the Cox proportional hazards regression model is robust against such deviations from the model assumptions.^22 34^ Our HR estimates were calculated using a robust bootstrapping procedure to provide reliable and internally validated results. We believe that because of the robustness of the model and the model specification, our HRs lie close to their true values.

### Future work

Replicating this study in other settings would offer the opportunity to validate the results from this study. Future analyses should directly investigate the implied relationship between inpatient capacity and patient time in the ED, suggested by our findings. Staffing data could be included in further analyses, fully accounting for staffing levels and staff seniority during the strike measures to align with previous work demonstrating an effect of staff seniority on mortality.^35^ There is evidence that strikes do not impact mortality, but this and other outcomes would be useful to include in future work.^36^ Inpatient occupancy as well as numbers and details of investigations may also offer insights into the efficiency of the ED operations during the strikes. Despite there being no official statistics on changes in capacity during strike days, the reported cancellations of elective care in inpatient departments^25^ would mean some additional capacity was made available. While there may not be formal changes to processes during strikes, it would be useful to determine whether staff behaviour changes could effectively alter them. For example, staff understanding of how strikes affect services may influence decisions about which service a patient is referred to. Further investigation of such possibilities would be valuable. Further investigation into the long-term effects of strikes should also be investigated. EDs may see increased attendances in the days around a strike. Postponement of elective activity may also result in emergency attendances in the longer term due to conditions going untreated. To fully understand our results, we suggest a qualitative study to gain perspectives from patients, clinicians and other members of the NHS workforce affected by the strikes.

## Conclusion

There are relatively few studies of the effect of strikes on healthcare systems. Strikes provide an interesting counterfactual to the usual operation of NHS services. This study found improved flow through the ED during certain strike days, which we infer is largely due to improved inpatient capacity. This suggests that during non-strike periods, patient flow through NHS EDs can be improved by expanding capacity and efficiently discharging medically fit patients.

## Supplementary material

10.1136/emermed-2024-214452 online supplemental file 1

## Data Availability

No data are available.
