# Supplementary material for: Evaluating the impact of NHS strikes on patient flow through emergency departments
Source: Emerg Med J. 2025 Dec 25;43(3):e214452. doi: 10.1136/emermed-2024-214452 (PMC13018738; doi:10.1136/emermed-2024-214452)
Supplement: online supplemental file 1 [file emermed-43-3-s001.docx]

**Supplementary Materials - Evaluating the Impact of NHS Strikes on Patient Flow through Emergency Departments**

Table of Contents

[Strike Dates 2](#_Toc208745608)

[Sample Flow Diagram 4](#_Toc208745609)

[Referred to Service Categories 4](#_Toc208745610)

[Model Covariates 4](#_Toc208745611)

[Exploratory Analysis Results ED1 6](#_Toc208745612)

[Kaplan Meier Exploratory Analysis Results ED1 6](#_Toc208745613)

[Tests for Proportional Hazards ED1 8](#_Toc208745614)

[Cox-Proportional Hazards Results ED1 10](#_Toc208745615)

[ED2 Analysis 12](#_Toc208745616)

[Additional Analysis 14](#_Toc208745617)

## Strike Dates

Strike days that directly impacted the emergency department were investigated. Regional strikes outside of Lancashire and strikes for specific specialties were not accounted for. The online resource strikecalendar.co.uk was used to derive the dates. These dates were cross-referenced with a Wikipedia database of NHS strikes since the start of 2022, no discrepancies were found (1,2) a table of strike dates and associated striking groups used in the analysis, the table can be found in the supplementary materials.

Truth table for which strike dates are classed as which strike.

Table S 1: Strike dates included in the analysis with associated strike groups.

|  | Nurse strike | Ambulance strike | Junior doctor strike | Consultant strike |
| --- | --- | --- | --- | --- |
| 15/12/2022 | TRUE | FALSE | FALSE | FALSE |
| 20/12/2022 | TRUE | FALSE | FALSE | FALSE |
| 21/12/2022 | FALSE | TRUE | FALSE | FALSE |
| 11/01/2023 | FALSE | TRUE | FALSE | FALSE |
| 18/01/2023 | TRUE | FALSE | FALSE | FALSE |
| 19/01/2023 | TRUE | FALSE | FALSE | FALSE |
| 23/01/2023 | FALSE | TRUE | FALSE | FALSE |
| 06/02/2023 | TRUE | FALSE | FALSE | FALSE |
| 07/02/2023 | TRUE | FALSE | FALSE | FALSE |
| 10/02/2023 | FALSE | TRUE | FALSE | FALSE |
| 01/03/2023 | TRUE | FALSE | FALSE | FALSE |
| 02/03/2023 | TRUE | FALSE | FALSE | FALSE |
| 03/03/2023 | TRUE | FALSE | FALSE | FALSE |
| 13/03/2023 | FALSE | FALSE | TRUE | FALSE |
| 14/03/2023 | FALSE | FALSE | TRUE | FALSE |
| 15/03/2023 | FALSE | FALSE | TRUE | FALSE |
| 20/03/2023 | FALSE | TRUE | FALSE | FALSE |
| 11/04/2023 | FALSE | FALSE | TRUE | FALSE |
| 12/04/2023 | FALSE | FALSE | TRUE | FALSE |
| 13/04/2023 | FALSE | FALSE | TRUE | FALSE |
| 14/04/2023 | FALSE | FALSE | TRUE | FALSE |
| 15/04/2023 | FALSE | FALSE | TRUE | FALSE |
| 14/06/2023 | FALSE | FALSE | TRUE | FALSE |
| 15/06/2023 | FALSE | FALSE | TRUE | FALSE |
| 16/06/2023 | FALSE | FALSE | TRUE | FALSE |
| 13/07/2023 | FALSE | FALSE | TRUE | FALSE |
| 14/07/2023 | FALSE | FALSE | TRUE | FALSE |
| 15/07/2023 | FALSE | FALSE | TRUE | FALSE |
| 16/07/2023 | FALSE | FALSE | TRUE | FALSE |
| 17/07/2023 | FALSE | FALSE | TRUE | FALSE |
| 18/07/2023 | FALSE | FALSE | TRUE | FALSE |
| 20/07/2023 | FALSE | FALSE | FALSE | TRUE |
| 21/07/2023 | FALSE | FALSE | FALSE | TRUE |
| 22/07/2023 | FALSE | FALSE | FALSE | TRUE |
| 11/08/2023 | FALSE | FALSE | TRUE | FALSE |
| 12/08/2023 | FALSE | FALSE | TRUE | FALSE |
| 13/08/2023 | FALSE | FALSE | TRUE | FALSE |
| 14/08/2023 | FALSE | FALSE | TRUE | FALSE |
| 24/08/2023 | FALSE | FALSE | FALSE | TRUE |
| 25/08/2023 | FALSE | FALSE | FALSE | TRUE |
| 19/09/2023 | FALSE | FALSE | FALSE | TRUE |
| 20/09/2023 | FALSE | FALSE | TRUE | TRUE |
| 21/09/2023 | FALSE | FALSE | TRUE | FALSE |
| 22/09/2023 | FALSE | FALSE | TRUE | FALSE |
| 02/10/2023 | FALSE | FALSE | TRUE | TRUE |
| 03/10/2023 | FALSE | FALSE | TRUE | TRUE |
| 04/10/2023 | FALSE | FALSE | TRUE | TRUE |
| 20/10/2023 | FALSE | FALSE | TRUE | FALSE |
| 21/10/2023 | FALSE | FALSE | TRUE | FALSE |
| 22/10/2023 | FALSE | FALSE | TRUE | FALSE |
| 03/01/2024 | FALSE | FALSE | TRUE | FALSE |
| 04/01/2024 | FALSE | FALSE | TRUE | FALSE |
| 05/01/2024 | FALSE | FALSE | TRUE | FALSE |
| 06/01/2024 | FALSE | FALSE | TRUE | FALSE |
| 07/01/2024 | FALSE | FALSE | TRUE | FALSE |
| 08/01/2024 | FALSE | FALSE | TRUE | FALSE |
| 25/02/2024 | FALSE | FALSE | TRUE | FALSE |
| 26/02/2024 | FALSE | FALSE | TRUE | FALSE |
| 27/02/2024 | FALSE | FALSE | TRUE | FALSE |
| 28/02/2024 | FALSE | FALSE | TRUE | FALSE |
| 15/12/2022 | TRUE | FALSE | FALSE | FALSE |
| 20/12/2022 | TRUE | FALSE | FALSE | FALSE |
| 21/12/2022 | FALSE | TRUE | FALSE | FALSE |
| 11/01/2023 | FALSE | TRUE | FALSE | FALSE |

Sample Flow Diagram


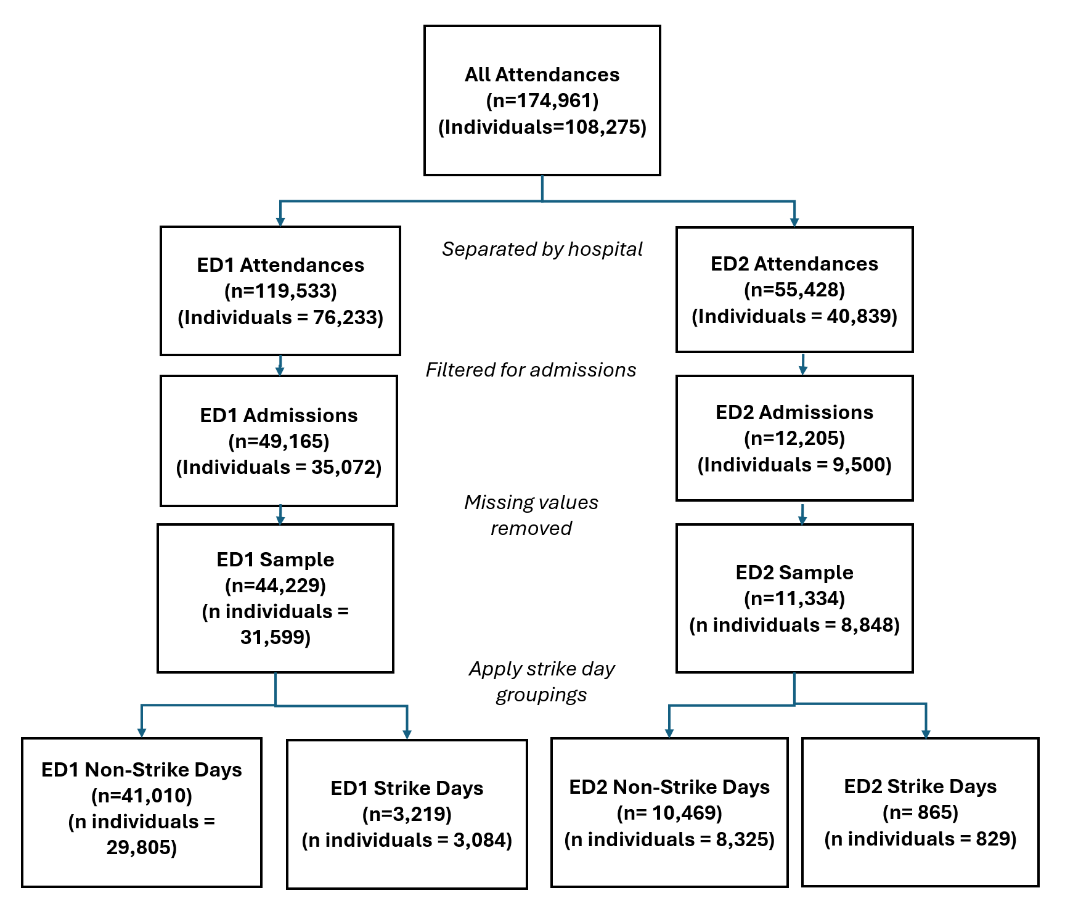


Figure S 1: Flow diagram demonstrating the data specification process with sample and cohort sizes.

## Referred to Service Categories

Referred to Service categories included; Ear, nose and throat, General medical, Gynaecological, Neurosurgical, Orthopaedic, Other, Paediatric, Plastic Surgery, Stroke, Surgical, Urology.

Referral destination categories in the ‘Other’ class are Geriatric, Cardiology, Nephrology, Obstetrics, Clinical oncology, Ophthalmology, Clinical oncology, Neurology, Rehabilitation, Endocrinology.

## Model Covariates

Here we provide more detail on the covariates of the model.

**ED Factors** – Covariates relating to the ED at any given point in the study period.

Linear time effect – An integer that increases by one each day of the study period, to capture any linear changes in outcome over time.

Seasonal effects – harmonic pair – The linear time covariate sine and cosine transformed with a period of one year, into two covariates to capture seasonal variations throughout the course of the year. ($\sin\left( \frac{2\pi}{365}t \right) , \cos\left( \frac{2\pi}{365}t \right)$).

Time of day – harmonic pair – The hour of the day (0-23) sine and cosine transformed with a period of one year, into two covariates to capture the variation over the course of the day.

Weekend – Binary indicator variable. One for Saturdays and Sundays, zero otherwise.

‘Heat’ - A weighted count of patients in the ED, calculated at time of arrival. Weights correspond to urgency of patient recorded in ED data. Weights are assigned to the categories in descending urgency, the most urgent presentations are given 5x the weight of the least urgent, the second most is given 4x and so on, for each of the five categories. In this Each presentation contributes the current heat from when they arrive at the ED until they leave the ED. Standardised for use in the model.

**Patient Presentation Factors** – Covariates relating to each patient presentation.

Urgency of presentation – The recorded urgency of a presentation recorded in ED data. 1-highest urgency, 5-least urgent. This corresponds to the Initial Assessment Triage Category(3).

Referred to service – The recorded service a patient presentation was referred to at the end of their time in ED. See Referred to Service Categories for available categories.

**Patient Demographic Factors** – Covariates relating to a patient’s demographic information at time of arrival to ED.

Age – The recorded age of a patient in the ED data. Grouped into categories (0-18], (18, 25], (25, 30], (30, 45], (45, 65], (65, 80], ≥ 80.

Ethnicity – The recorded ethnicity of a patient. Categories include; ‘White’, ‘Asian or Asian British’, ‘Black or Black British’, ‘Mixed’, ‘Other Ethnic Groups’, ‘Not stated’.

Gender – The recorded gender of a patient. ‘Male’, ‘Female’ or ‘Not known’.

## Exploratory Analysis Results ED1


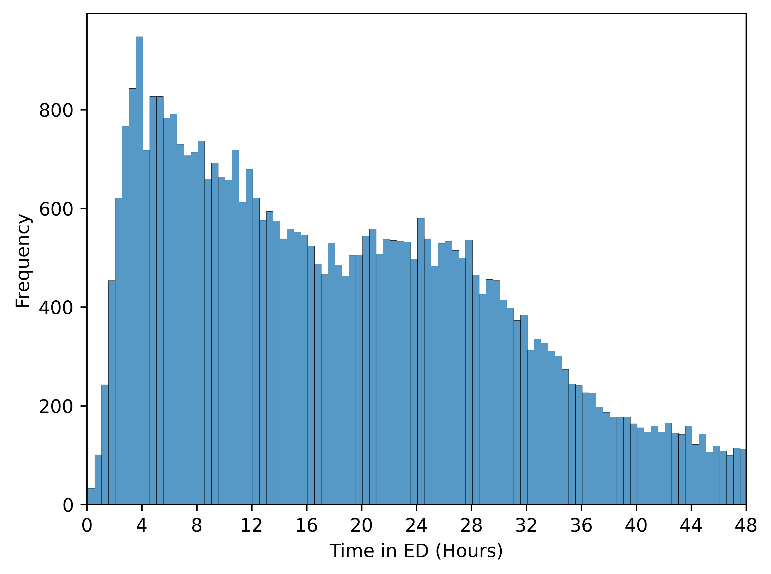


Figure S 2: Histogram of admitted patients' time spent in ED.

In Figure S 2 we see a high, widespread distribution of patients’ time in ED given subsequent admission (EDGSA). This is because we are looking at just patients who were admitted since the start of 2022. Admitted patients generally are in ED for longer, and patient time in EDGSA has increased over time, so this is a subset of attendances with a high time in EDGSA.


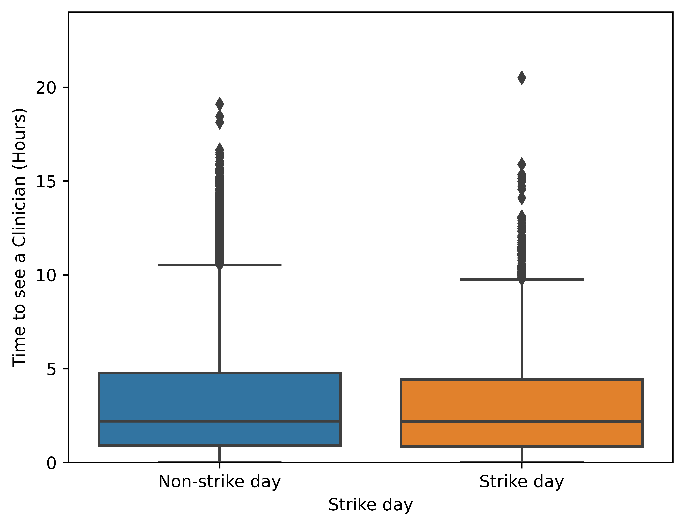


Figure S 3: Box plot of time taken to be seen by a clinician, separated by strike days and non-strike days.

Figure S 3 shows the difference in time taken to be seen by a clinician. As mentioned in the main manuscript, the KM test suggests that these two groups are different. However, in this figure it is likely that this is due to the large sample size (every admission within this period) and possibly outlying values where patients have waited longer than 24 hours.

## Kaplan Meier Exploratory Analysis Results ED1

We produced Kaplan-Meier plots to demonstrate time in EDGSA curves, separated by categorical variables. Figure S 4 - Figure S 7


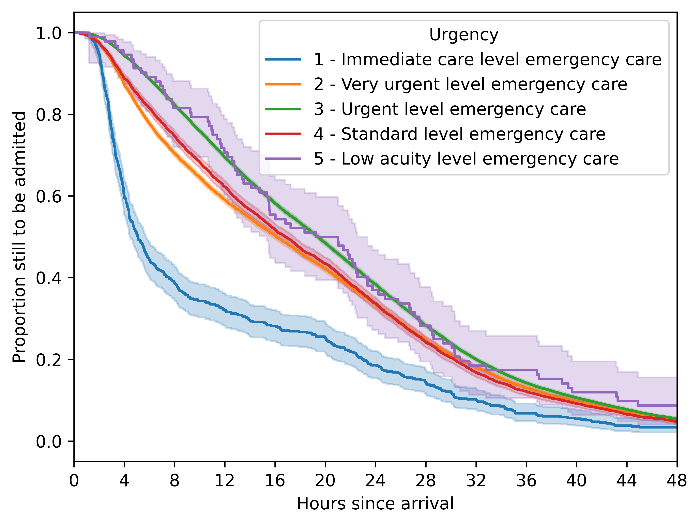


Figure S 4: Kaplan-Meier time in EDGSA curves stratified by urgency.


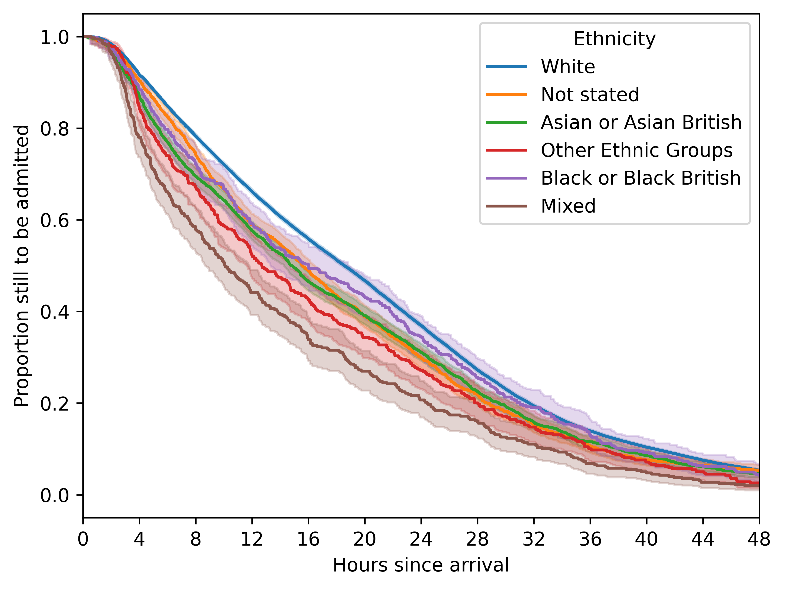


Figure S 5: Kaplan-Meier “time in EDGSA” curves stratified by ethnicity.


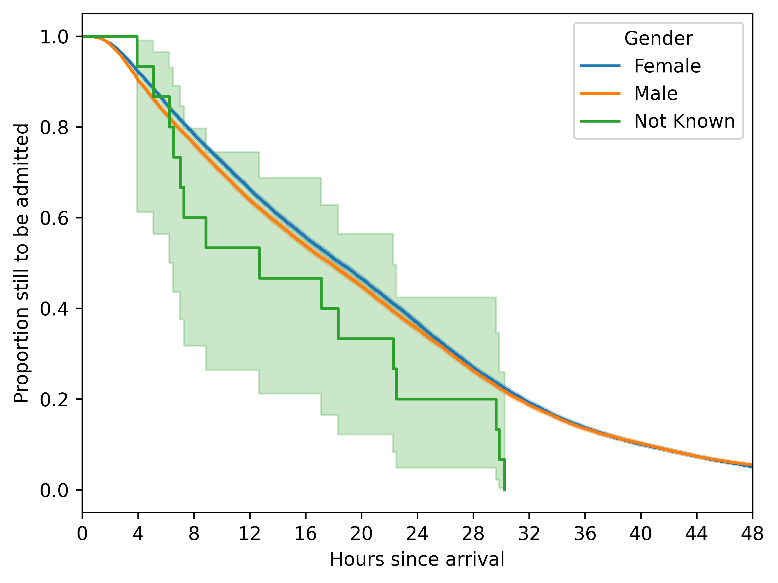


Figure S 6: Kaplan-Meier “time in EDGSA” curves stratified by gender.


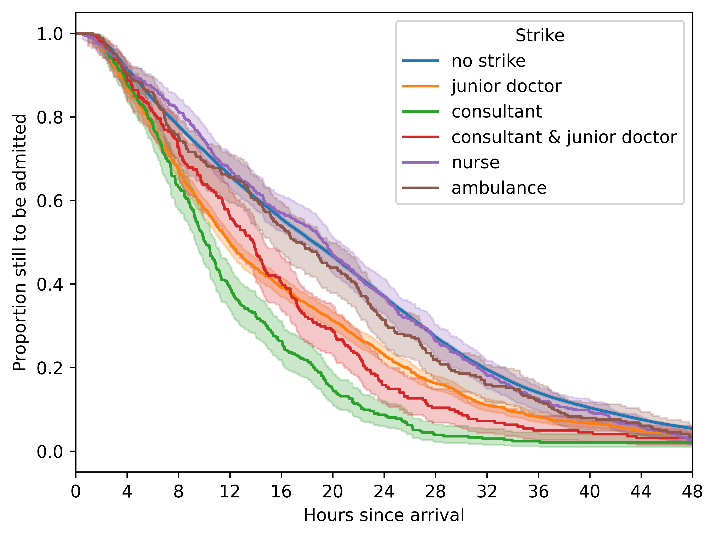


Figure S 7: Kaplan-Meier “time in EDGSA” curves stratified by strike type.

## Tests for Proportional Hazards ED1

**Log-log plots**

We can assess the proportional hazards assumption by plotting the transformed time in EDGSA function $S\left( t \right)$ against the log-transformed time. For the proportional hazards assumption to hold, the curves should be roughly parallel.


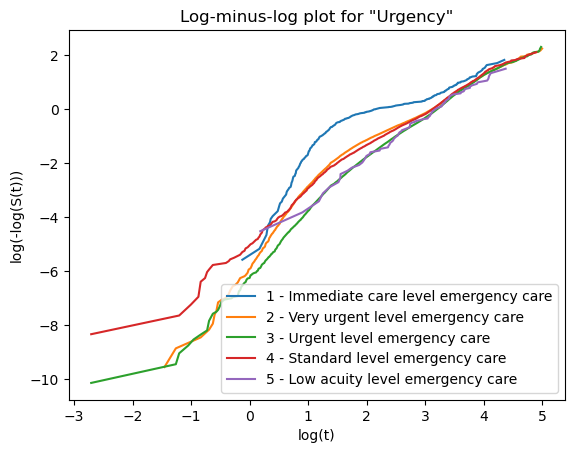


Figure S 8: Log-minus log plot of Cox Proportional Hazard functions stratified by the Urgency variable.


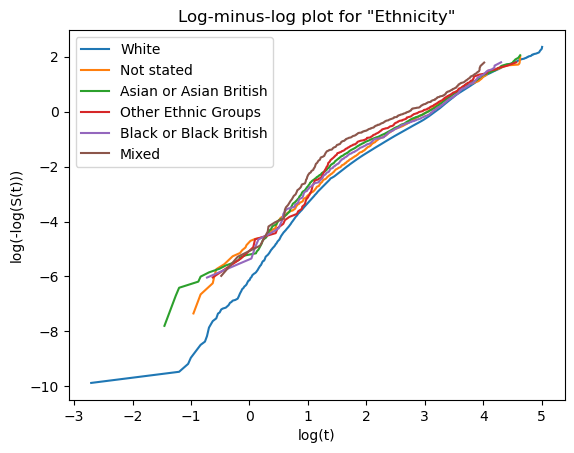


Figure S 9: Log-minus log plot of Cox Proportional Hazard functions stratified by the Ethnicity variable.


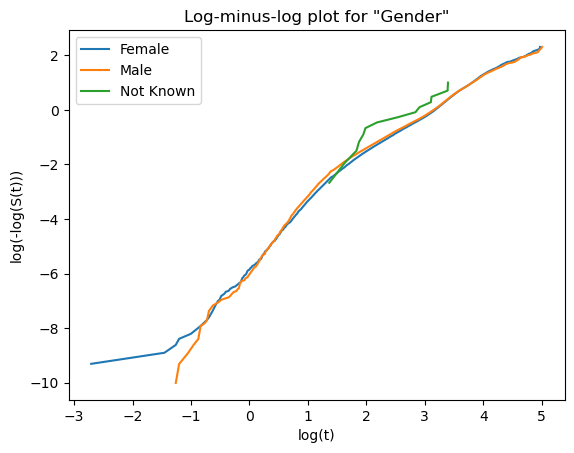


Figure S 10: Log-minus log plot of Cox Proportional Hazard functions stratified by the Gender variable.


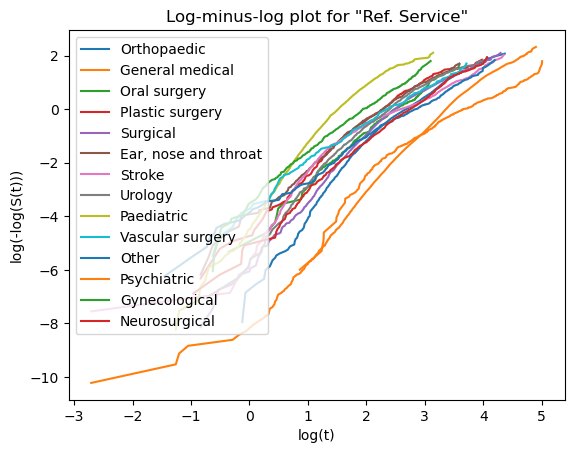


Figure S 11: Log-minus log plot of Cox Proportional Hazard functions stratified by the Referred to Service variable.


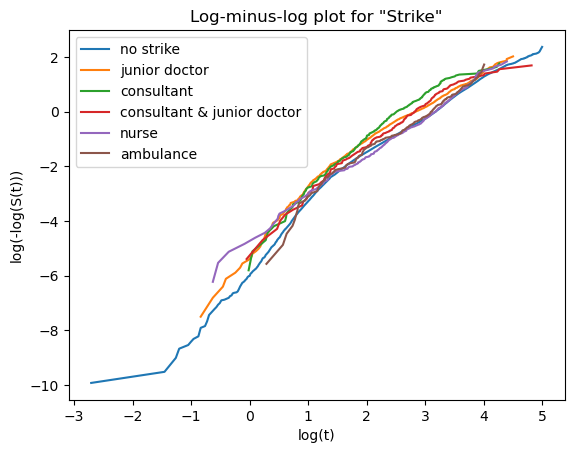


Figure S 12: Log-minus log plot of Cox Proportional Hazard functions stratified by the Strike Type variable.

## Cox-Proportional Hazards Results ED1

Table S 2: Full coefficients, and corresponding hazards ratios, for the Cox-proportional hazards model.

| Variable | Level | Coefficient | Hazard Ratio | 95% CI | | p-value |
| --- | --- | --- | --- | --- | --- | --- |
| Linear time trend | - | 0.009 | 1.009 | 1.007 | 1.01 | <0.0001 |
| Yearly harmonic (sine) | - | 0.014 | 1.014 | 0.998 | 1.03 | 0.088488 |
| Yearly harmonic term (cosine) | - | -0.053 | 0.948 | 0.933 | 0.964 | <0.0001 |
| Daily harmonic term (sine) | - | -0.006 | 0.994 | 0.979 | 1.009 | 0.445167 |
| Daily harmonic term (cosine) | - | 0.007 | 1.007 | 0.992 | 1.023 | 0.339748 |
| ED heat | - | -0.016 | 0.984 | 0.983 | 0.985 | 0 |
| Urgency | 2 | -0.407 | 0.666 | 0.592 | 0.749 | <0.0001 |
| Urgency | 3 | -0.458 | 0.632 | 0.562 | 0.711 | <0.0001 |
| Urgency | 4 | -0.399 | 0.671 | 0.594 | 0.757 | <0.0001 |
| Urgency | 5 – least urgent | -0.48 | 0.619 | 0.49 | 0.781 | <0.0001 |
| Referred to Service | General medical | -1.119 | 0.326 | 0.31 | 0.344 | 0 |
| Referred to Service | Oral surgery | 0.182 | 1.199 | 1.009 | 1.426 | 0.038942 |
| Referred to Service | Plastic surgery | 0.474 | 1.607 | 1.356 | 1.903 | <0.0001 |
| Referred to Service | Surgical | 0.149 | 1.161 | 1.081 | 1.246 | <0.0001 |
| Referred to Service | Ear, nose and throat | 0.526 | 1.692 | 1.484 | 1.929 | <0.0001 |
| Referred to Service | Stroke | -0.129 | 0.879 | 0.802 | 0.964 | 0.006367 |
| Referred to Service | Urology | 0.409 | 1.506 | 1.349 | 1.681 | <0.0001 |
| Referred to Service | Paediatric | 1.759 | 5.806 | 5.364 | 6.286 | 0 |
| Referred to Service | Vascular surgery | 0.386 | 1.472 | 1.204 | 1.798 | 0.000157 |
| Referred to Service | Other | -0.422 | 0.656 | 0.573 | 0.751 | <0.0001 |
| Referred to Service | Psychiatric | -2.242 | 0.106 | 0.091 | 0.124 | <0.0001 |
| Referred to Service | Gynaecology | 1.088 | 2.969 | 2.622 | 3.363 | <0.0001 |
| Referred to Service | Neurosurgical | -0.194 | 0.824 | 0.748 | 0.906 | <0.0001 |
| Weekend | True | -0.34 | 0.712 | 0.693 | 0.731 | <0.0001 |
| Age |  | -0.008 | 0.992 | 0.992 | 0.993 | <0.0001 |
| Ethnicity | Not Stated | -0.018 | 0.983 | 0.916 | 1.054 | 0.622487 |
| Ethnicity | Asian or Asian British | 0.018 | 1.018 | 0.974 | 1.065 | 0.434191 |
| Ethnicity | Other Ethnic Groups | -0.061 | 0.941 | 0.839 | 1.055 | 0.293996 |
| Ethnicity | Black or Black British | 0.011 | 1.011 | 0.909 | 1.125 | 0.838245 |
| Ethnicity | Mixed | 0.042 | 1.043 | 0.934 | 1.165 | 0.451928 |
| Gender | Male | -0.003 | 0.997 | 0.975 | 1.018 | 0.767701 |
| Gender | Not known | 0.326 | 1.385 | 0.785 | 2.443 | 0.260395 |
| Strike type | Junior doctor | 0.117 | 1.124 | 1.059 | 1.194 | 0.000138 |
| Strike type | Consultant | 0.249 | 1.283 | 1.113 | 1.478 | 0.0006 |
| Strike type | Consultant & junior doctor | 0.099 | 1.105 | 0.918 | 1.33 | 0.293484 |
| Strike type | Nurse | 0.115 | 1.122 | 1.024 | 1.229 | 0.0134 |
| Strike type | Ambulance | 0.073 | 1.076 | 0.947 | 1.222 | 0.259944 |


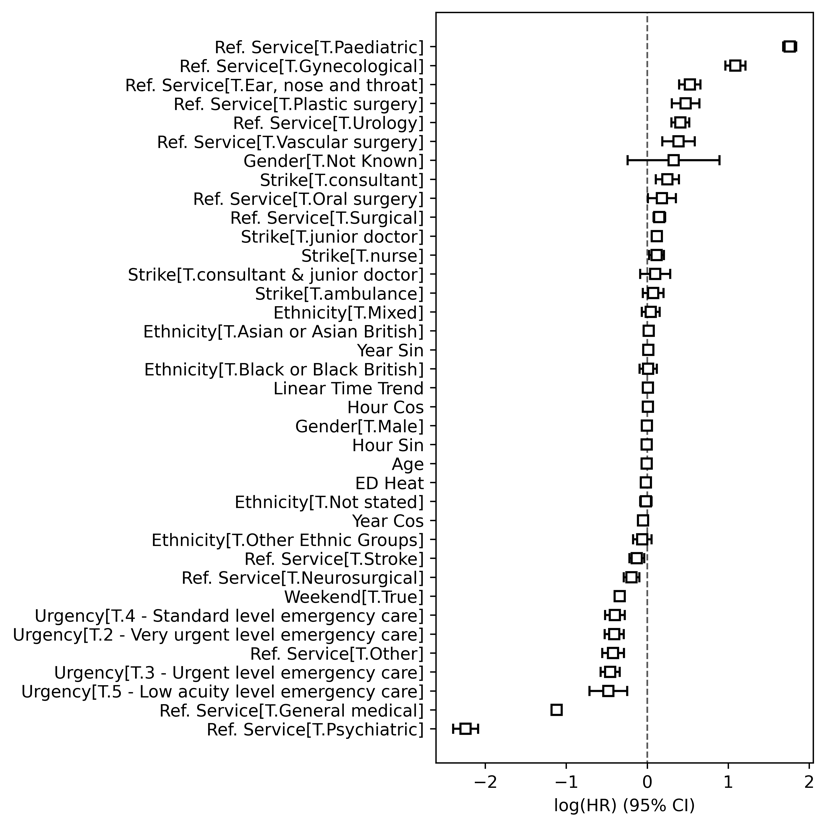


Figure S 13: Forrest plot for all variables included in the model.

## ED2 Analysis

**Exploratory Kaplan-Meier Curves**

The model for ED2 did not include referred to service, due to low variance between categories and therefore convergence issues during model fitting. This similarity between referred to service time in EDGSA curves can be seen in the Kaplan-Meier plot in Figure S 14.


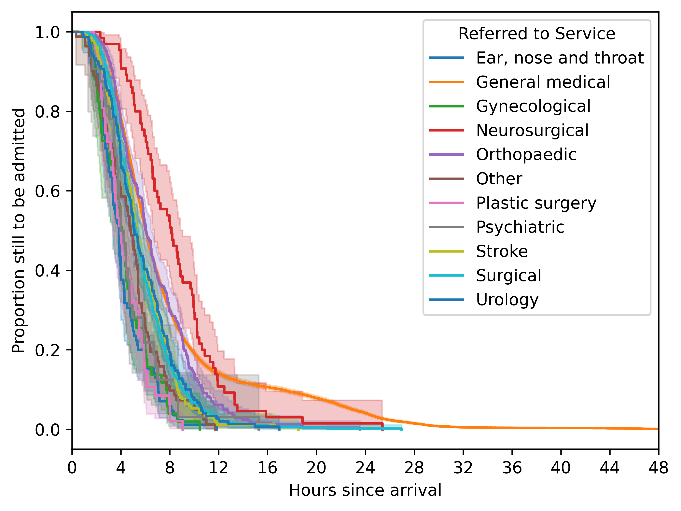


Figure S 14: ED2 - Kaplan-Meier curves of admitted patients' time in EDGSA, separated by referred to service they are admitted to. The shaded area around each line represents the 95% confidence interval (CI) for the KM estimate at each point.

**Cox-Proportional Hazards Model**

The coefficients and hazard ratios for the strikes at ED2 are shown in Table S 3. The results from ED2 are less consistent. None of the strike types appear to have a significant impact on patient flow through the hospital, with junior doctor and both consultant and junior doctor strikes appearing to show improved flow, and the others slowing flow. The entire model coefficients and hazard ratios can be found in Table S 4.

Table S 3:ED2 - Coefficients and hazard ratios for the strike variables in the Cox-proportional hazards model. Here, higher hazard refers to a higher likelihood of being admitted into the hospital.

| Strike Type | Coefficient | Hazard Ratio | 95% CI | | p-value | |
| --- | --- | --- | --- | --- | --- | --- |
| Baseline – No strike |  |  | - | - | | - |
| Junior doctor strike | 0.085 | 1.089 | 1.034 | 1.147 | | 0.001342* |
| Consultant strike | -0.077 | 0.925 | 0.842 | 1.018 | | 0.110248 |
| Consultant and junior doctor strike | 0.328 | 1.388 | 1.124 | 1.713 | | 0.002309* |
| Nurse strike | -0.238 | 0.788 | 0.615 | 1.01 | | 0.060099 |
| Ambulance strike | -0.012 | 0.988 | 0.843 | 1.158 | | 0.883701 |
| ** Statistically significant* | | | | | | |

The resulting fitted time in EDGSA curves from the Cox model are shown in Figure S 15. In this case it is visible that time in EDGSA reduces for some strikes and increases for others.


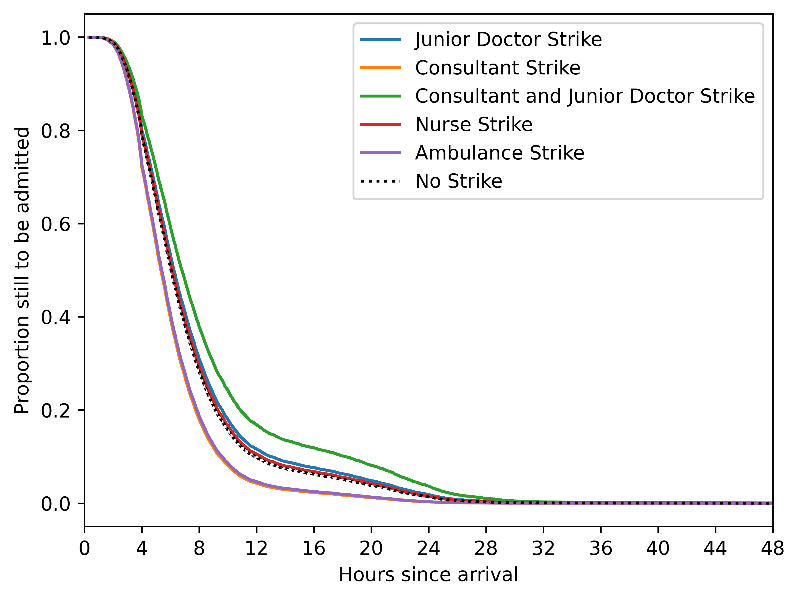


Figure S 15: ED2 - Fitted Cox-regression time in EDGSA curves for each of the different strike types in the analysis.

Full model coefficients for ED2 can be found in Table S 4.

Table S 4: ED2 – Full coefficients, and corresponding hazards ratios, for the Cox-proportional hazards model.

| Variable | Level | Coefficient | Hazard Ratio | 95% CI | | p-value |
| --- | --- | --- | --- | --- | --- | --- |
| Linear time trend |  | -0.039 | 0.961 | 0.959 | 0.964 | <0.0001 |
| Yearly harmonic (sine) |  | -0.047 | 0.954 | 0.926 | 0.982 | 0.001556 |
| Yearly harmonic term (cosine) |  | -0.191 | 0.826 | 0.803 | 0.85 | <0.0001 |
| ED heat |  | -0.021 | 0.979 | 0.977 | 0.981 | <0.0001 |
| Urgency | 2 | 0.02 | 1.02 | 0.677 | 1.537 | 0.924668 |
| Urgency | 3 | -0.076 | 0.926 | 0.616 | 1.394 | 0.713926 |
| Urgency | 4 | -0.105 | 0.901 | 0.597 | 1.358 | 0.616966 |
| Urgency | 5 – least urgent | 0.064 | 1.066 | 0.539 | 2.108 | 0.854914 |
| Age |  | -0.004 | 0.996 | 0.995 | 0.998 | <0.0001 |
| Ethnicity | Not Stated | -0.088 | 0.916 | 0.82 | 1.023 | 0.120668 |
| Ethnicity | Asian or Asian British | 0.117 | 1.124 | 0.922 | 1.371 | 0.248239 |
| Ethnicity | Other Ethnic Groups | -0.091 | 0.913 | 0.694 | 1.201 | 0.513445 |
| Ethnicity | Black or Black British | -0.156 | 0.855 | 0.602 | 1.214 | 0.382266 |
| Ethnicity | Mixed | -0.22 | 0.802 | 0.525 | 1.226 | 0.30895 |
| Gender | Male | 0.052 | 1.053 | 1.013 | 1.094 | 0.008402 |
| Gender | Not known | -0.049 | 0.952 | 0.462 | 1.962 | 0.894035 |
| Strike type | Junior doctor | 0.085 | 1.089 | 1.034 | 1.147 | 0.001342 |
| Strike type | Consultant | -0.077 | 0.925 | 0.842 | 1.018 | 0.110248 |
| Strike type | Consultant & junior doctor | 0.328 | 1.388 | 1.124 | 1.713 | 0.002309 |
| Strike type | Nurse | -0.238 | 0.788 | 0.615 | 1.01 | 0.060099 |
| Strike type | Ambulance | -0.012 | 0.988 | 0.843 | 1.158 | 0.883701 |

## Additional Analysis

**ED Heat ED1**

In an additional addional analysis, we tested fitting the models without the ‘ED heat’ variable because there is a possibility it is on the causal pathway between the variable or interest (strike day) and the outcome (time in EDGSA). The results of the modelling are included in Table S 5. More hazard ratios are significant and have a greater magnitude than those in the main analysis. However, we consider the main analysis more robust because despite the fact there might be a small influence of heat on time in EDGSA it is an important upstream covariate that needs to be adjusted for in the analysis.

Table S 5: Modelling output results of the additional analysis, with the ED heat variable removed.

| Strike Type | Coefficient | Hazard Ratio | 95% CI | | p-value | |
| --- | --- | --- | --- | --- | --- | --- |
| Baseline – No strike |  |  | - | - | | - |
| Junior doctor strike | 0.304 | 1.355 | 1.279 | 1.436 | | <0.0001* |
| Consultant strike | 0.607 | 1.834 | 1.594 | 2.111 | | <0.0001* |
| Consultant and junior doctor strike | 0.27 | 1.31 | 1.099 | 1.562 | | 0.002566* |
| Nurse strike | 0.189 | 1.208 | 1.108 | 1.317 | | <0.0001* |
| Ambulance strike | 0.223 | 1.25 | 1.103 | 1.416 | | 0.000479* |
| ** Statistically significant* | | | | | | |

**References**

1. UK Strike Action Calendar [Internet]. [cited 2024 Apr 22]. Available from: https://www.strikecalendar.co.uk/

2. 2022–present National Health Service strikes. In: Wikipedia [Internet]. 2024 [cited 2024 Apr 22]. Available from: https://en.wikipedia.org/w/index.php?title=2022%E2%80%93present_National_Health_Service_strikes&oldid=1220068455

3. A AND E INITIAL ASSESSMENT TRIAGE CATEGORY [Internet]. [cited 2025 Sep 14]. Available from: https://archive.datadictionary.nhs.uk/DD%20Release%20September%202020/attributes/a_and_e_initial_assessment_triage_category.html
